# In this month’s Bulletin

**DOI:** 10.2471/BLT.18.000318

**Published:** 2018-03-01

In editorials this month, Shelly Chadha et al. (146) highlight future requirements for a public health response to hearing loss. Viroj Tangcharoensathien et al. (147) announce an upcoming theme issue on the prevention and control of noncommunicable diseases. Carlos Correa (148) comments on the use of flexibilities in the Agreement on Trade-Related Aspects of Intellectual Property Rights. 

Sima Barmania reports on architectural features of a medical research centre in Australia and a hospital in Norway, both designed to maximize the health of people who use them (151–152). Carol Brayne talks to Tatum Anderson about research on the prevention and epidemiology of dementia (153–154).

## Sri Lanka

### Preventing hospital readmission

Lelwala Guruge Thushani Shanika et al. (155–164) observe the difference made by ward-based clinical pharmacists.

## Islamic Republic of Iran

### Unseen consequences of contaminated opioids

Talat Ghane et al. (165–172) collate reports of lead toxicity.

## Barbados, Belgium, Brunei Darussalam, Chile, Dominica, Fiji, Finland, France, Hungary, Kiribati, Mauritius, Mexico, Norway, Saint Vincent and the Grenadines, Samoa, Saudi Arabia, Spain, Tonga, Vanuatu 

### Improving the food supply for population health

Anne Marie Thow et al. (201–210) examine fiscal policy measures designed to prevent non-communicable diseases.

## Canada, Guatemala, Italy, Kenya, Thailand, United Kingdom of Great Britain and Northern Ireland

### Applying a One-Health approach

Martin Hitziger et al. (211–218) trace implementation through the policy-making cycle. 

## Bangladesh, Côte d’Ivoire, Ethiopia, Ghana, India, Malawi, Nigeria, Uganda, United Republic of Tanzania

### Improving health service provision for mothers and babies

Abosede Adeniran et al. (222–224) present a partnership for maternal, newborn and child health.

### Detailing the scope of primary health care

Sandeep P. Kishore et al. (219–221) consider what’s needed for effective cardiovascular disease control.

### How to manage latent tuberculosis?

Ann Jagger et al. (173–184) survey national policies from 148 countries. 

### Mitigating the impact of trade agreements

Ellen F.M. 't Hoen et al. (185–193) review countries’ use of flexibilities intended to ensure access to medicines.

### Tuberculosis and antimicrobial resistance

Rumina Hasan et al. (194–200) proposes integration of control programmes.

